# Quality-adjusted time without symptoms or toxicity analysis of nivolumab plus chemotherapy versus chemotherapy alone for the management of previously untreated patients with advanced gastric cancer, gastroesophageal junction cancer, or esophageal adenocarcinoma

**DOI:** 10.1007/s10120-023-01372-7

**Published:** 2023-03-21

**Authors:** Daniel Lin, Hiep Nguyen, Ruchit Shah, Yao Qiao, John Hartman, Ryan Sugarman

**Affiliations:** 1 Thomas Jefferson University, 1025 Walnut Street, Suite 700 College Building, Philadelphia, PA 19107 USA; 2 Bristol Myers Squibb, Princeton, NJ USA; 3 Previously Employed at OPEN Health, Bethesda, MD USA; 4 Memorial Sloan Kettering, New York, NY USA

**Keywords:** Nivolumab, Gastric cancer, Quality-adjusted survival, Quality of life, Quality-adjusted time without symptoms or toxicity

## Abstract

**Background:**

The phase 3 CheckMate 649 established superior overall survival of nivolumab in combination with chemotherapy (NIVO + chemo) compared with chemotherapy (chemo) alone as a first-line treatment for patients with Her2-negative advanced gastric cancer, gastroesophageal junction cancer, and esophageal adenocarcinoma (GC/GEJC/EAC). This post hoc trial analysis aimed to evaluate the benefit of NIVO + chemo using quality-adjusted time without symptoms or toxicity (Q-TWiST) to further account for quality of life (QoL) in different health states depending on disease progression and treatment toxicity.

**Methods:**

Using data from CheckMate 649, we evaluated the quality-adjusted survival gain associated with NIVO + chemo compared with chemo alone among all randomized patients and repeated similar analyses among those with programmed cell death-ligand 1 (PD-L1) combined positive score (CPS) ≥ 5. Relative Q-TWiST gains of ≥ 10% were predefined as clinically important.

**Results:**

In all randomized patients, those receiving NIVO + chemo had a mean Q-TWiST gain of 1.8 (95% CI 0.9, 2.7) months compared with those receiving chemo alone. The relative Q-TWiST gain was estimated to be 12.8%. Patients with PD-L1 CPS ≥ 5 had greater quality-adjusted survival gain from NIVO + chemo with an estimated Q-TWiST gain of 2.8 (95% CI 1.5, 4.1) months, representing a relative gain of 20.6%. Subgroup analyses and sensitivity analyses with various QoL utility values yielded consistent findings in favor of NIVO + chemo compared with chemo alone. Q-TWiST gain from NIVO + chemo increased with longer duration of follow-up.

**Conclusions:**

NIVO + chemo was associated with a statistically significant and clinically important gain in quality-adjusted survival compared with chemo alone among previously untreated patients with advanced GC/GEJC/EAC.

## Introduction

Gastroesophageal cancers, including gastric cancer (GC), gastroesophageal junction cancer (GEJC) and esophageal adenocarcinoma (EAC), remain a leading cause of cancer-related deaths worldwide [1, 2]. GC, GEJC, and EAC share similar molecular profiles and have shown comparable clinical outcomes with chemotherapy in the advanced disease setting [3, 4, 5, 6]. These cancers are often diagnosed at advanced stages and are thus associated with a poor prognosis, with an estimated median survival of less than 1 year among those receiving chemotherapy [7, 8, 9]. Advanced gastroesophageal cancer has typically been treated with sequential lines of chemotherapy, starting with platinum and fluoropyrimidine as a standard first-line therapy [7, 9]. In the USA, however, 25% of patients with advanced or metastatic GC or GEJC remain untreated, and only approximately 50% of patients who receive first-line therapy go on to receive subsequent treatment [10]. Therefore, there is a strong need for improved therapies in this patient population.

Nivolumab, a programmed cell death (PD)-1 inhibitor, is the first immunotherapy approved by the US Food and Drug Administration (FDA) in combination with fluoropyrimidine- and platinum-containing chemotherapy for the treatment of patients with advanced or metastatic GC/GEJC/EAC, regardless of PD-ligand 1 (PD-L1) expression status, based on results from the CheckMate 649 trial (CM 649; NCT02872116) [11, 12]. CM 649 was a multicenter, randomized, open-label, phase 3 clinical trial conducted to evaluate nivolumab in combination with chemotherapy (NIVO + chemo) as a first-line therapy for advanced GC/GEJC/EAC compared with chemotherapy (chemo) alone [11, 13]. NIVO + chemo demonstrated superior overall survival (OS) compared with chemo alone, both in all randomized patients and among those with a PD-L1 combined positive score (PD-L1 CPS) ≥ 5 [11].

Although no new safety signals were identified with NIVO + chemo, a higher proportion of patients receiving NIVO + chemo reported grade 3–4 adverse events (AEs) compared with those receiving chemo (59% vs. 44%) [11]. Thus, there is a need to better understand the risk–benefit trade-off of NIVO + chemo versus chemo in this patient population, in the context of its clinical benefit. Additionally, given that treatment for advanced GC is typically administered until disease progression or unacceptable toxicity, quality of life (QoL) in relation to disease symptoms and treatment toxicities should also be incorporated into treatment evaluation. This need to better assess the value of treatments incorporating risk–benefit trade-offs, QoL data, and patient preference has been a major focus of oncology societies and regulatory agencies over the past several years [14, 15, 16, 17, 18, 19].

The quality-adjusted time without symptoms or toxicity (Q-TWiST) methodology provides a comprehensive framework for treatment comparison that accounts for both the quantity and quality of survival time [20]. It estimates net health benefits by partitioning survival time into distinct health states and accounts for treatment efficacy as well as toxicity [20]. This Q-TWiST analysis was undertaken to evaluate the quality-adjusted survival gain associated with NIVO + chemo compared with chemo among patients enrolled in CM 649.

## Methods

### Data source and study population

This study was a post hoc analysis of data from the CM 649 trial (12 months minimum follow-up; 33 months maximum follow-up; July 10, 2020, database lock) [11]. A total of 789 and 792 patients were randomized to the NIVO + chemo and chemo arms, respectively [11]. Chemo was administered as CAPOX (capecitabine and oxaliplatin) every 3 weeks or FOLFOX (fluorouracil, leucovorin, and oxaliplatin) every 2 weeks [11]. Patients randomized to NIVO + chemo received nivolumab at 360 mg every 3 weeks (with CAPOX) or 240 mg every 2 weeks (with FOLFOX) [11]. A total of 955 patients had a PD-L1 CPS ≥ 5, among whom 473 received NIVO + chemo and 482 received chemo. All patients continued treatment for 2 years or until disease progression, unacceptable toxicity, or withdrawal of consent. The analyses described below were performed both in the overall population and among the subgroup of patients with CPS ≥ 5.

### Statistical analysis

#### Q-TWiST method

In Q-TWiST analyses, survival time is partitioned into three distinct health states: time without disease progression or symptoms of toxicity (TWiST), time with toxicity before disease progression (TOX), and time after disease progression (REL).

The model included only the more severe toxicities (i.e., grade 3 or 4) because they were the events considered most likely to have greater impact on a patient’s QoL. Therefore, all grade 3 or 4 events attributable to either NIVO + chemo or chemo, according to the National Cancer Institute Common Terminology Criteria for Adverse Events version 4.0., were included in the analysis, apart from those starting after progression. The type, date of onset, and date of resolution of each toxicity were recorded prospectively as a part of the standard procedure in conducting a randomized phase 3 trial. The duration of an AE was defined as the duration of time between the start and end date (resolution date) of the AE if the AE had resolved by the time of progression or death. If the AE had not been resolved by the time of progression or death, then the date of progression was used in the place of the AE end date. Disease progression in CM 649 measured by the date of first documented tumor progression or death was assessed by blinded independent central review using RECIST version 1.1 criteria [11].

The Q-TWiST for each treatment group was calculated as the weighted sum of mean time spent in each health state where the state-specific QoL utilities served as the weights (i.e., Q-TWiST = *U*_TWiST_ × TWiST + *U*_TOX_ × TOX + *U*_REL_ × REL). The base case analysis relied on the following assumptions: (1) the analysis used the conventional utility values of *U*_TWiST_ = 1, *U*_TOX_ = 0.5, and *U*_REL_ = 0.5 [19]; (2) *U*_TOX_ was set at 0.5 regardless of AE severity or type; (3) the end date for each AE was the earliest of AE resolution date, disease progression, death, or censoring; (4) TOX duration was the total number of days spent with AEs of interest before disease progression, if observed, or the end of study follow-up, where a day with AE was counted only once even if there were multiple events documented on the same day; and (5) to calculate the total number of days in TOX, all days with AEs before progression or end of study follow-up in the absence of progression were grouped together, regardless of whether they were consecutive events or not.

To calculate the mean duration spent in each health state, we constructed Kaplan–Meier (K–M) curves for TOX, progression-free survival (PFS), and OS and estimated the mean durations of TOX, PFS, and OS using area under the curve (AUC). TWiST time was computed as the difference in AUC between PFS and TOX, whereas REL time was calculated as the difference in AUC between OS and PFS. All restricted means were evaluated at the maximum follow-up of 33 months, which was the longest time of follow-up from CM 649 trial at the time of publication [11].

Finally, the Q-TWiST gain was calculated as the Q-TWiST among individuals receiving NIVO + chemo subtracted by the Q-TWiST among those receiving chemo. The relative Q-TWiST gain was computed as the Q-TWiST gain divided by the mean OS among those receiving chemo. Relative Q-TWiST gains above 10% and 15% were considered clinically important and clearly clinically important, respectively, based on criteria established by Revicki et al. [13]. To evaluate the precision of the estimated restricted mean durations in each health state, Q-TWiST, and Q-TWiST gain, we used a non-parametric bootstrap method with 1000 bootstrapped samples of trial patients generated with replacement. The 95% confidence intervals (CIs) were estimated using the corresponding 2.5th and 97.5th percentiles.

#### Threshold analysis, sensitivity analysis, and subgroup analysis

As the perceived state-specific QoL utilities may vary for different individuals, we performed a threshold analysis allowing the utility values of TOX and REL to vary over the full range between 0 and 1 to quantify treatment benefits with respect to individual preferences. Additionally, we conducted a sensitivity analysis reflecting the health-specific QoL in our study population, where the utility values were computed as the average EQ-5D-3L scores reported during time in each health state in the CM 649 trial. To evaluate how the Q-TWiST gain changes over time, we performed a sensitivity analysis to estimate Q-TWiST gains at various follow-up times, including 3, 6, 9, 12, 15, 18, 21, 24, 27, and 30 months, in addition to the maximum follow-up duration. Second, to explore the robustness of our findings to a different definition of AE, we conducted another sensitivity analysis where AEs of interest included not only grade 3 and 4 AEs, but also grade 2 AEs that lasted for 28 days or longer. Lastly, we performed additional Q-TWiST analyses in prespecified subgroups from CM 649 among all randomized patients and patients with PD-L1 CPS ≥ 5.

Additionally, CM 649 analyzed multiple prespecified subgroups with predetermined subcategories for each subgroup. These subgroups and a priori defined categories included age (< 65 years, ≥ 65 years), gender (female, male), race (Asian, White, other), region (Asia, United States/Canada, rest of the world), Eastern Cooperative Oncology Group (ECOG) performance status (0, 1), PD-L1 expression on tumor cells (≥ 1%, < 1%), primary tumor location (GC, GEJC, EAC), liver metastases (yes, no), signet ring cell carcinoma (yes, no), microsatellite instable (MSI) status (microsatellite stable [MSS], microsatellite stable-high [MSI-H]), and chemo regimen initiated (CAPOX, FOLFOX).

## Results

### Summary of CM 649 trial results

The CM 649 trial included patients (*N* = 1581) aged 18 years or older with previously untreated advanced or metastatic GC/GEJC/EAC. Approximately, three-quarters (1206 [76%] of 1581) of patients were non-Asian and most had GC (1110 [70%] of 1581), while 16% had GEJ cancer and 13% had EAC. At a 12-month minimum follow-up for 1581 randomized patients, the CM 649 trial found that NIVO + chemo provided a significant and clinically meaningful OS (HR 0.80 [99.3% CI 0.68–0.94]) benefit vs chemo in all randomized GC/GEJC/EAC patients. In patients with PD-L1 CPS ≥ 5, the treatment benefit of NIVO + chemo vs chemo was even greater for OS (0.71 [98.4% CI 0.59–0.86]). [11] OS benefit was observed in multiple prespecified subgroups, as well. Grade 3–4 treatment-related AEs were reported in 59% (NIVO + chemo) and 44% (chemo) of patients with the most common for the NIVO + chemo cohort being neutropenia (15%) and anemia (6%); for chemo patients, they were neutropenia (11–13%) and diarrhea (3–4%). Further details on patient and disease baseline characteristics and study results are provided in the main publication for CM 649 [11].

### Q-TWiST base case analysis

In the overall population, patients receiving NIVO + chemo had significantly longer TWiST by 1.4 (95% CI 0.5, 2.4) months and longer TOX by 0.6 (95% CI 0.4, 0.9) months, on average (Table 1), compared with patients on chemo alone. The two arms did not show a significant difference in REL. Based on conventionally used utility values of 1, 0.5, and 0.5 for TWiST, TOX, and REL, respectively, Q-TWiST was estimated to be 13.0 (95% CI 12.3, 13.7) months among those receiving NIVO + chemo and 11.2 (95% CI 10.6, 11.8) months among those receiving chemo. The gain in Q-TWiST associated with the addition of NIVO was estimated to be 1.8 (95% CI 0.9, 2.7) months, and the corresponding relative Q-TWiST gain was estimated to be 12.8%, which was considered clinically important. Similarly, among patients with PD-L1 CPS ≥ 5, NIVO + chemo was associated with a statistically significant and clinically important Q-TWiST gain of 2.8 (95% CI 1.5, 4.1) months (relative Q-TWiST gain: 20.6%) compared with chemo alone.

**Table 1:** Mean duration of each health state and Q-TWiST (95% CI)

Health state	NIVO + chemo	Chemotherapy alone	Difference
*Overall population*			
TOX	1.3 (1.1, 1.5)	0.7 (0.6, 0.8)	0.6 (0.4, 0.9)
TWiST	10.0 (9.3, 10.7)	8.6 (7.8, 9.2)	1.4 (0.5, 2.4)
REL	4.7 (4.0, 5.3)	4.6 (4.1, 5.3)	0.0 (-0.9, 0.9)
Q-TWiST	13.0 (12.3, 13.7)	11.2 (10.6, 11.8)	1.8 (0.9, 2.7)
*PD-L1 CPS* ≥ *5*			
TOX	1.3 (1.1, 1.6)	0.7 (0.5, 0.8)	0.7 (0.4, 0.9)
TWiST	10.6 (9.6, 11.6)	8.3 (7.4, 9.2)	2.3 (1.0, 3.7)
REL	4.9 (4.1, 5.7)	4.6 (3.9, 5.3)	0.3 (-0.8, 1.4)
Q-TWiST	13.7 (12.8, 14.6)	11.0 (10.1, 11.8)	2.8 (1.5, 4.1)

*CI* confidence interval, *CPS* combined positive score, *NIVO + chemo* nivolumab in combination with chemotherapy, *Q-TWiST* quality-adjusted time without symptoms or toxicity, *REL* time after progression, *TOX* time with grade 3 or higher adverse event before disease progression, *TWiST* time without disease progression or symptoms of toxicity

### Q-TWiST threshold analysis, sensitivity analyses, and subgroup analyses

**Fig. 1:**
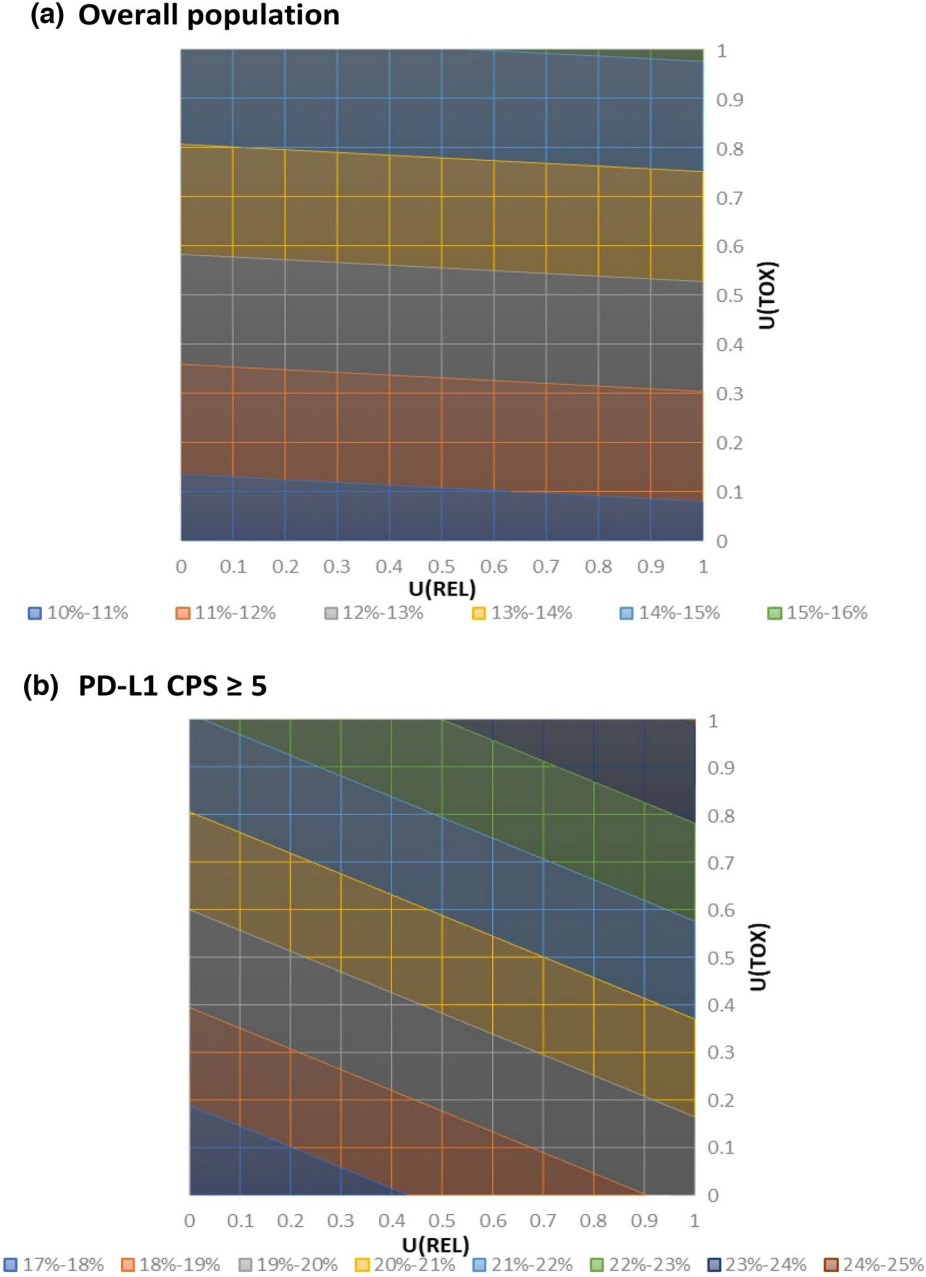
Threshold analysis. **a** Overall population, **b** PD-L1 CPS ≥ 5. *CPS* combined positive score, *REL* time after progression, *TOX* time with grade 3 or higher adverse event before disease progression

In a threshold analysis with varying utility values of TOX and REL, the Q-TWiST gains associated with NIVO + chemo compared with chemo ranged from 1.4 (95% CI 0.5, 2.4) to 2.1 (95% CI 1.1, 3.2) months and remained statistically significant with all combinations of utility values (Table 2). The relative Q-TWiST gain was greater than 10%, which was considered clinically meaningful across the full range of TOX and REL utility values (Table 2, Fig. 1a). This includes the use of EQ-5D-3L values reported in the trial population, where the Q-TWiST gain of NIVO + chemo was 1.8 (95% CI 0.9, 2.7) months and the relative gain was nearly 13%, representing a clinically important increase. Similarly, among patients with PD-L1 CPS ≥ 5, findings were robust across the full range of utilities of TOX and REL (Table 2, Fig. 1b), including utility values derived from the EQ-5D-3L scores (Q-TWiST gain: 2.6 [95% CI 1.5, 3.7] months; relative gain: 19.1%).

**Table 2:** Threshold analysis

Q-TWiST utility values	NIVO + chemo Q-TWiST (95% CI)	Chemo Q-TWiST (95% CI)	Q-TWiST Gain (95% CI)	Relative Q-TWiST Gain
*Overall population*				
U(TOX) = 0, U(REL) = 0	10.0 (9.3, 10.7)	8.6 (7.8, 9.2)	1.4 (0.5, 2.4)	10.4%
U(TOX) = 0, U(REL) = 0.5	12.4 (11.7, 13)	10.9 (10.2, 11.5)	1.5 (0.6, 2.4)	10.5%
U(TOX) = 0, U(REL) = 1	14.7 (13.9, 15.5)	13.2 (12.5, 13.9)	1.5 (0.5, 2.5)	10.6%
U(TOX) = 0.5, U(REL) = 0	10.7 (9.9, 11.4)	8.9 (8.2, 9.6)	1.8 (0.8, 2.7)	12.6%
U(TOX) = 0.5, U(REL) = 0.5	13.0 (12.3, 13.7)	11.2 (10.6, 11.8)	1.8 (0.9, 2.7)	12.8%
U(TOX) = 0.5, U(REL) = 1	15.3 (14.6, 16.2)	13.5 (12.9, 14.3)	1.8 (0.8, 2.9)	12.9%
U(TOX) = 1, U(REL) = 0	11.3 (10.5, 12.1)	9.3 (8.5, 9.9)	2.1 (1.1, 3.1)	14.9%
U(TOX) = 1, U(REL) = 0.5	13.7 (12.9, 14.4)	11.6 (10.9, 12.2)	2.1 (1.1, 3.0)	15.0%
U(TOX) = 1, U(REL) = 1	16.0 (15.2, 16.8)	13.9 (13.2, 14.6)	2.1 (1.1, 3.2)	15.1%
*PD-L1 CPS* ≥ *5*				
U(TOX) = 0, U(REL) = 0	10.6 (9.6, 11.6)	8.3 (7.4, 9.2)	2.3 (1.0, 3.7)	17.1%
U(TOX) = 0, U(REL) = 0.5	13.1 (12.2, 14)	10.6 (9.8, 11.5)	2.5 (1.2, 3.8)	18.1%
U(TOX) = 0, U(REL) = 1	15.5 (14.5, 16.5)	12.9 (12.0, 13.9)	2.6 (1.2, 4.0)	19.2%
U(TOX) = 0.5, U(REL) = 0	11.3 (10.2, 12.3)	8.7 (7.7, 9.5)	2.6 (1.3, 4.1)	19.5%
U(TOX) = 0.5, U(REL) = 0.5	13.7 (12.8, 14.6)	11.0 (10.1, 11.8)	2.8 (1.5, 4.1)	20.6%
U(TOX) = 0.5, U(REL) = 1	16.2 (15.1, 17.2)	13.3 (12.3, 14.2)	2.9 (1.5, 4.4)	21.6%
U(TOX) = 1, U(REL) = 0	12.0 (10.9, 12.9)	9.0 (8.0, 9.9)	3.0 (1.7, 4.4)	21.9%
U(TOX) = 1, U(REL) = 0.5	14.4 (13.4, 15.3)	11.3 (10.4, 12.1)	3.1 (1.8, 4.5)	23.0%
U(TOX) = 1, U(REL) = 1	16.8 (15.8, 17.8)	13.6 (12.6, 14.5)	3.3 (1.8, 4.7)	24.1%

*CI* confidence interval, *CPS* combined positive score, *NIVO + chemo* nivolumab in combination with chemotherapy, *Q-TWiST* quality-adjusted time without symptoms or toxicity, *REL* time after progression, *TOX* time with grade 3 or higher adverse event before disease progression

**Fig. 2:**
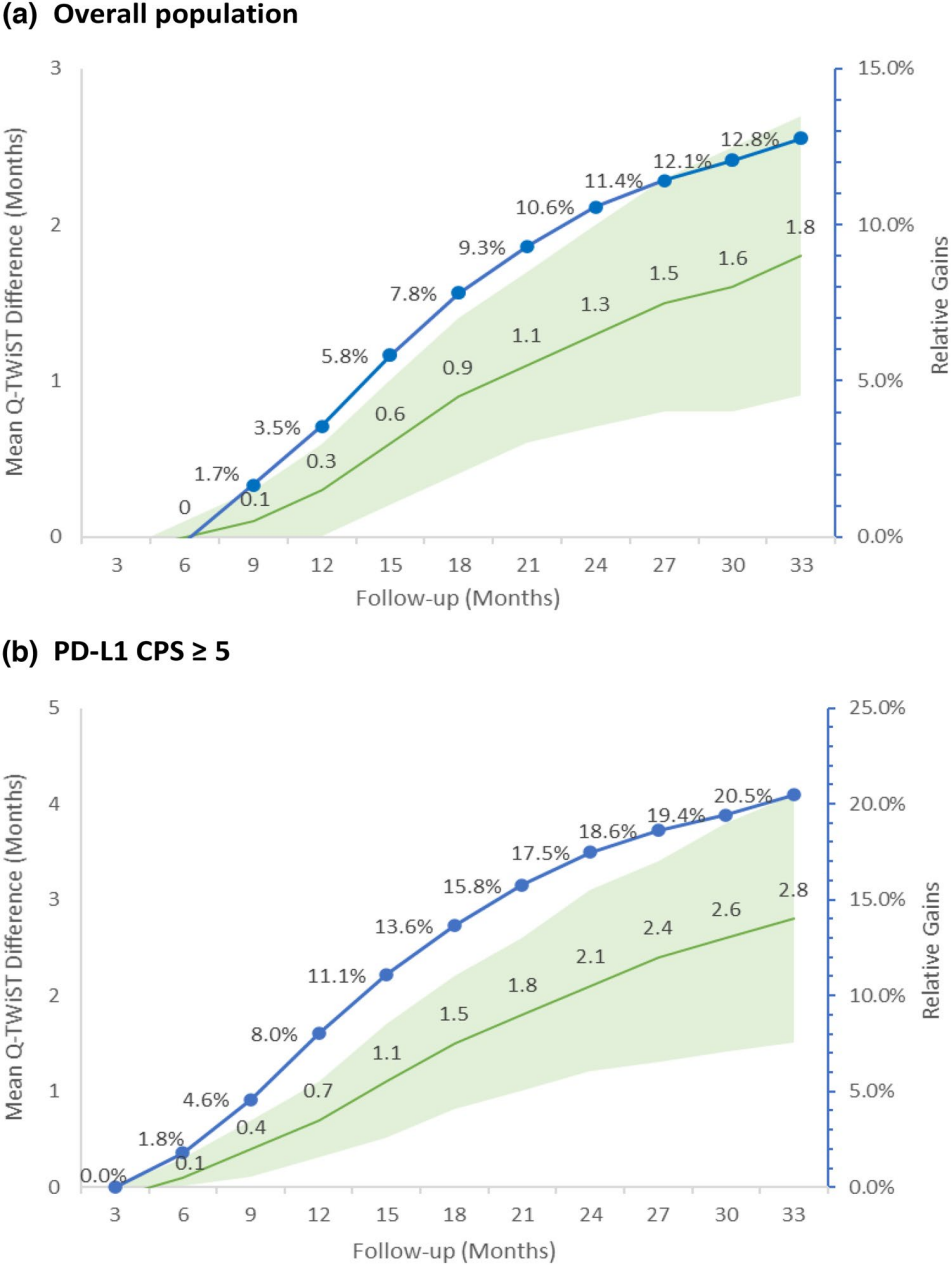
Sensitivity analysis at different follow-up time points. **a** Overall population, **b** PD-L1 CPS ≥ 5. *CPS* combined positive score, *Q-TWiST* quality-adjusted time without symptoms or toxicity

Sensitivity analyses at various follow-up durations demonstrated a positive relationship between the amount of follow-up time and Q-TWiST gains for NIVO + chemo compared with chemo alone among all randomized patients (Fig. 2a) and patients with CPS ≥ 5 (Fig. 2b). Also, expanding the definition of TOX to include grade 2 AEs lasting for at least 28 days lowered the Q-TWiST gains of NIVO + chemo to 1.3 (95% CI 0.5, 2.2) months among all randomized patients, but the results remained significant; this sensitivity analysis yielded consistent findings favoring NIVO + chemo over chemo alone among patients with PD-L1 CPS ≥ 5 (Q-TWiST gain: 2.2 [95% CI 1.0, 3.4] months).

**Fig. 3:**
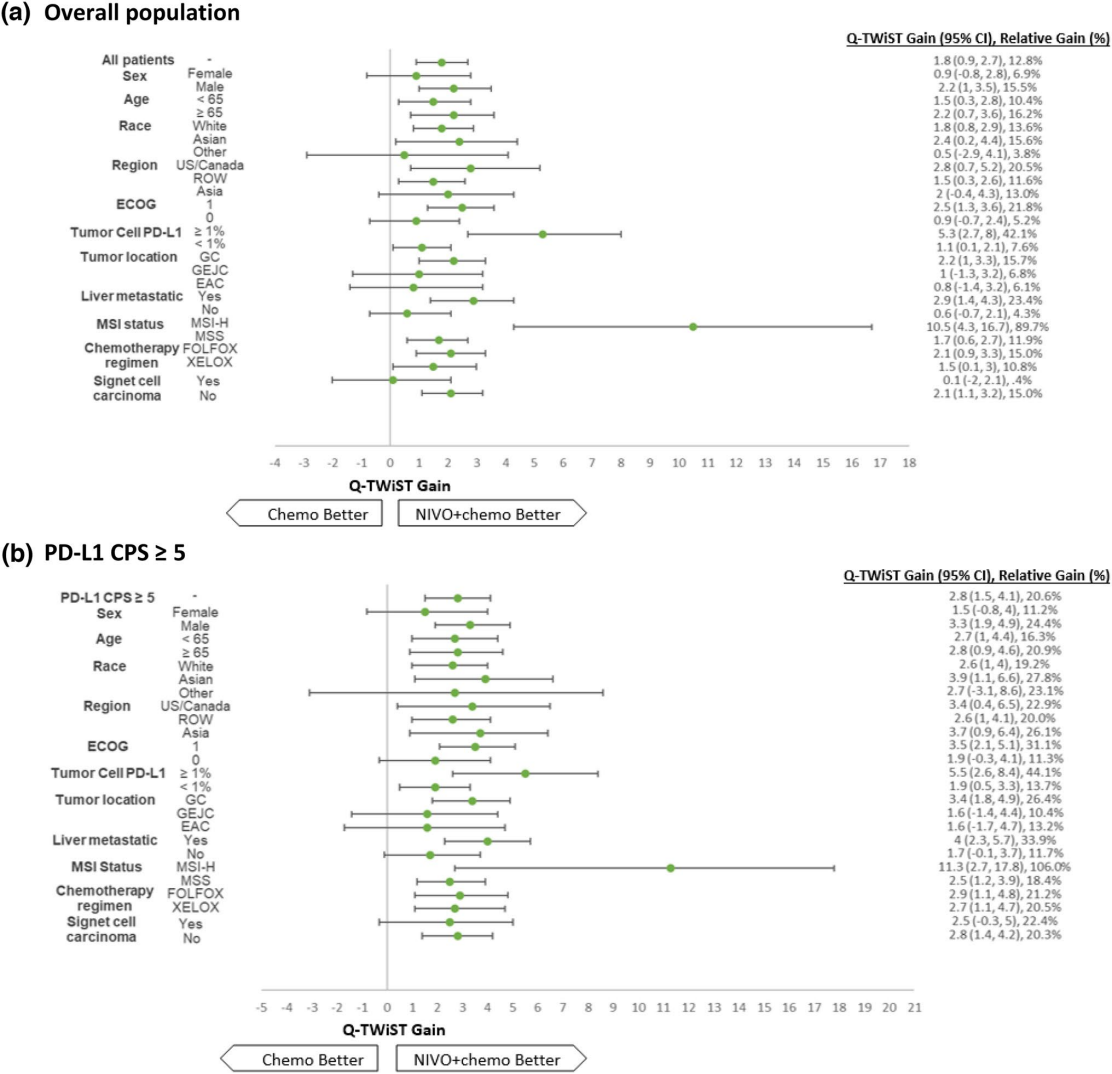
Q-TWiST gain (95% CI) and the relative Q-TWiST gain in subgroup analyses (patient subgroups of interest predefined in CM 649). **a** Overall population, **b** PD-L1 CPS ≥ 5. *CI* confidence interval, *CPS* combined positive score, *EAC* esophageal adenocarcinoma, *ECOG* Eastern Cooperative Oncology Group, *FOLFOX* leucovorin, fluorouracil, and oxaliplatin, *GC* gastric cancer, *GEJC* gastroesophageal junction cancer, *MSI* microsatellite instable, *MSI-H* microsatellite stable-high, *MSS* microsatellite stable, *PD-L1* programmed cell death-ligand 1, *Q-TWiST* quality-adjusted time without symptoms or toxicity, *ROW* rest of the world, *US* United States, *XELOX* capecitabine and oxaliplatin

The results of the subgroup analyses among all randomized patients are summarized in Fig. 3a. Compared with the chemo alone arm, the NIVO + chemo arm demonstrated a greater Q-TWiST in all subgroups. The greatest quality-adjusted survival gain with NIVO + chemo was observed among patients with MSI-H (10.5 [95% CI 4.3, 16.7] months), followed by those with tumor cell PD-L1 ≥ 1% (5.3 [95% CI 2.7, 8] months). Similarly, Q-TWiST difference favored NIVO + chemo over chemo alone across all subgroups among those with PD-L1 CPS ≥ 5 (Fig. 3b).

## Discussion

Using data from the phase 3 CM 649 trial in patients with HER2-negative, advanced GC/GEJC/EAC, this post hoc analysis found a statistically significant gain in quality-adjusted survival by 1.8 (95% CI 0.9, 2.7) months when NIVO + chemo was compared with chemo alone in previously untreated patients with advanced GC/GEJC/EAC. This gain was considered clinically important based on an estimated Q-TWiST gain of 12.8%. In patients with PD-L1 CPS ≥ 5, the quality-adjusted survival associated with the addition of nivolumab to chemo was estimated to be 2.8 (95% CI 1.5, 4.1) months, with a corresponding relative gain of 20.6%. The Q-TWiST gain also increased with longer duration of follow-up, suggesting the Q-TWiST gains of NIVO + chemo may increase even further with additional follow-up in the CM 649 trial. The pattern in favor of NIVO + chemo held true for various subgroups, with the greatest gain shown among patients with MSI-H, followed by the prespecified subgroup of patients with tumor cell PD-L1 ≥ 1%.

CM 649 is one of the largest clinical trials ever conducted among previously untreated patients with advanced GC/GEJC/EAC, with nearly 1600 patients randomly assigned to receive either NIVO + chemo or chemo [11]. This study established that NIVO + chemo was associated with superior OS versus chemo alone in this patient population. While the safety profiles were as expected, patients receiving NIVO + chemo, however, appeared more likely to report a grade 3–4 AE than those receiving chemo alone. In this Q-TWiST analysis, we reanalyzed the CM 649 trial data to take into consideration the potential negative impact on QoL due to these treatment-related AEs. Our findings suggest that after incorporating AE risk and accounting for patient QoL in different health states, NIVO + chemo remained the favored treatment option based on a significantly longer and clinically meaningful quality-adjusted survival time compared with chemo alone. This observation is consistent with the trend of improved health-related quality of life (HRQoL) exploratory endpoint in CM 649 for NIVO + chemo vs chemo [11]. This Q-TWiST analysis thus further supports the CM 649 trial findings by providing a risk–benefit perspective combining safety and efficacy endpoints.

Despite advances in the treatment of gastroesophageal cancers, they remain one of the highest causes of cancer death. As these cancers are often diagnosed at an advanced stage, the approval of immunotherapy in combination with chemotherapy provides additional therapeutic options for clinicians to help improve patient care. To our knowledge, prior to CM 649 trial, there had been no global studies reporting a median OS exceeding 1 year among patients with HER2-negative GC/GEJC/EAC in the first-line setting. Considering the poor prognosis of this patient population, a nearly 2-month survival gain that was quality-adjusted associated with NIVO + chemo reported in this study represents a meaningful improvement in patient care.

The present analysis utilized the Q-TWiST method that has been in use since the mid-1980s to evaluate the risk–benefit profile of competing interventions; however, there are certain limitations. First, the primary analysis considered only grade 3–4 AEs and did not account for grade 1–2 AEs, which may also impact QoL, especially if symptoms are ongoing. To fully quantify the benefit of therapies, we performed a sensitivity analysis incorporating grade 2 AEs lasting 28 days or longer, which yielded consistent findings to the primary analysis. Second, our analysis did not distinguish between AEs of different types and assumed the same QoL utilities for all AEs of interest. Third, certain drug-related effects may not be reportable as AEs but may affect patient QoL. We were unable to capture these potential events in this analysis. Fourth, the analysis did not include AEs that occurred after disease progression. Finally, the predefined relative Q-TWiST gains threshold of ≥ 10% in our study was met in some subgroups but not others, and multiple testing correction was not applied; hence, results from subgroups should be interpreted with caution.

Nonetheless, our study demonstrates several strengths. The analysis relies on a well-established method that was developed specifically to assess quality-adjusted survival in oncology. The Q-TWiST method has been applied in numerous treatment comparisons across multiple indications. Additionally, more than 50 such analyses in oncology have been published, including a study comparing trastuzumab plus chemotherapy versus chemotherapy alone as first-line treatment for advanced HER2-positive GC using data from the phase 3 ToGA (Trastuzumab for Gastric Cancer) trial [21]. These analyses serve as benchmarks that allow a comparison between our study findings and published results. For comparison, in prior analyses in HER2-positive patients with advanced gastric cancer, trastuzumab plus chemotherapy extended Q-TWiST by 2.42 months compared with chemotherapy alone[22], while recently trastuzumab deruxtecan was also estimated to extend quality-adjusted survival by 0.9 months compared to chemotherapy (6.6 vs 5.7 months) over 10 months of follow-up [23]. When benchmarked against previously published Q-TWiST analyses of multiple oncology therapies that were included in a benchmark review [20], the relative gain among all randomized patients in the CM-649 trial was higher than the majority (68%) of published results for oncology therapies (Fig. 1a). Lastly, this study performed multiple threshold, sensitivity, and subgroup analyses to explore the robustness of our findings.

In conclusion, our Q-TWiST analysis, which combines both efficacy and safety endpoints and accounts for patient QoL in different health states, demonstrated a statistically significant and clinically important gain in quality-adjusted survival of NIVO + chemo compared with chemo alone among previously untreated patients with advanced GC/GEJC/EAC. These Q-TWiST benefits were further increased in patients with tumors of higher PD-L1 expression (CPS ≥ 5). These results add to the findings from the CM 649 trial, supporting the use of NIVO + chemo as the standard of care for patients with advanced GC/GEJC/EAC.

## Figures and Tables

**Fig. 4:**
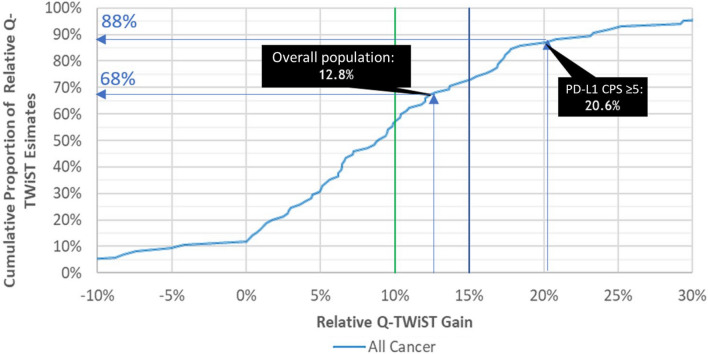
Cumulative proportion of relative Q-TWiST gain estimates associated with oncology therapies. *CPS* combined positive score, *Q-TWiST* quality-adjusted time without symptoms or toxicity

## Data Availability

The data analyzed during the current study are available upon request.

## Appendix

See Fig. 4.
